# Molecular Diagnosis of the Main Hemoparasites of Dairy Cows in the State of Ceará

**DOI:** 10.3390/genes15111369

**Published:** 2024-10-24

**Authors:** Gilderlândio Pinheiro Rodrigues, Beatriz Dantas Fernandes, Bruno Vinicios Silva de Araújo, Jaciara de Oliveira Jorge Costa, Milena Melo Silva, André de Macêdo Medeiros, Arlei Marcili, Juliana Fortes Vilarinho Braga, Michelly Fernandes de Macedo

**Affiliations:** 1 Laboratory of Diagnostics in Veterinary Clinical Pathology, Federal Rural University of the Semi-Arid, Mossoró 59625-900, RN, Brazil; gilpinheiromelo@gmail.com (G.P.R.); milenaameelo@hotmail.com (M.M.S.); 2 Laboratory of Reproductive Technologies and Innovations in Animal Models, Federal Rural University of the Semi-Arid, UFERSA, Mossoró 59625-900, RN, Brazil; beatrizdantasfernandes@gmail.com; 3 Laboratory of Morphophysiology Pharmacology, Federal Rural University of the Semi-Arid, Mossoró 59625-900, RN, Brazil; brunovinicios.araujo@hotmail.com; 4 Laboratory of Parasitic Diseases, Department of Preventive Veterinary Medicine and Animal Health, University of São Paulo, São Paulo 05508-220, SP, Brazil; jaciaraojcosta@gmail.com; 5 Department of Health Sciences I, Center for Biological and Health Sciences, Federal Rural University of the Semi-Arid, Mossoró 59625-900, RN, Brazil; andre.medeiros@ufersa.edu.br; 6 Public Health Program, Santo Amaro University, UNISA, São Paulo 04829-300, SP, Brazil; amarcili@prof.unisa.br; 7 Campus Profa Cinobelina Elvas, Federal University of Piauí, Bom Jesus 64900-000, PI, Brazil; juliana.braga@ufpi.edu.br

**Keywords:** anemia, babesiosis, polymerase chain reaction, trypanosomiasis

## Abstract

Background/Objectives: Trypanosomiasis and bovine babesiosis correspond to important diseases that cause great economic losses, but there are still no studies evaluating their occurrence in herds of dairy cattle in Ceará. The aim of this study was to perform molecular diagnosis of the main hemoparasites of dairy cows in the microregion of the central hinterland of Ceará. Methods: For the molecular diagnosis of parasites, genomic material was extracted and polymerase chain reaction directed to the cdCatL-like gene for *Trypanossoma vivax* and SS rRNA of *Babesia bigemina* and *Babesia. bovis* was performed. In addition, the mean corpuscular volume of the samples was evaluated. The data were statistically processed. Results: *T. vivax* was detected in 0.40% (1/246) of the samples, while *B. bigemina* and *B. bovis* were detected in 20.62% (33/160) and 11.87% (19/160) of the samples, respectively. It was found that there was a reduction in mean corpuscular volume in animals that presented with co-infection and those infected by *B. bovis* only, but not in those hosting *B. bigemina* alone. The variables “purchase of recent animals” and “tick control” had no association with or influence on *B. bovis* and/or *B. bigemina* infection. It was possible to identify epidemiologically important factors that may facilitate the transmission of trypanosoma to healthy animals, such as the recent purchase of animals and use of the same needle and syringe for oxytocin application. Conclusions: The pathogens studied were present in the evaluated population. Daily cow management practices can facilitate the transmission of the diseases they cause.

## 1. Introduction

Within any animal production system, one of the main objectives is to achieve good economic efficiency, which means that farmers must produce at a low cost to obtain a positive balance, with greater gains and returns on their investment. However, some diseases, such as parasitic infections, can limit and restrict these results; they are a major cause of economic loss in animal husbandry, especially in tropical areas and developing countries. As a result, the search for greater control of diseases and costs has become critical in dairy farming [1].

There is wide variation in the parasitic diseases, such as trypanosomiasis and babesiosis, that can affect herds with various impacts. Bovine trypanosomiasis is a disease caused by protozoa of the genus *Trypanosoma*, with wide distribution and economic importance in herds within Africa, Central America, and South America. In Brazil, outbreaks of *Trypanossoma vivax (T. vivax)* have already been reported in several states and studies have indicated a higher prevalence of the disease in herds in the country in the last decade [2,3].

The transmission of trypanosomiasis can occur biologically, through flies of the genus *Glossina* sp., also known as Tsé Tsé, or via the mechanical form—considered more relevant to Brazil—which occurs through hematophagous flies, such as tabanids, and especially the stable fly (*Stomoxys calcitrans*), contaminated fomites, the transplacental route, and, more recently, via the colostrum. In the semi-arid region of the Brazilian Northeast, the environment seems to be unfavorable for the development of vectors most of the year, and due to the long period of drought and high temperatures, animals are not infected and, therefore, do not develop active immunity. Thus, outbreaks and positive cases usually occur during the rainy season, when insects are more abundant [4,5,6].

Among the clinical symptoms of animals affected by parasites are fever, weight loss, anemia, hypoglycemia and neurological symptoms such as motor incoordination, temporary or permanent blindness and histopathological changes such as meningoencephalitis and encephalomalacia [4]. In addition, the disease can lead to greater economic losses due to the pronounced drop in milk production, abortion, the birth of weak calves, anomalous estrous and anestrous cycles when it affects females, as well as a loss of libido, delayed puberty, and inadequate semen quality in males [7].

Similarities in the clinical signs caused by *T. vivax* and other diseases, such as babesiosis, suggest that bovine trypanosomiasis may be subject to erroneous diagnoses or even underdiagnoses in the state of Ceará and in Brazil. The drugs commonly used for the treatment of babesiosis may aggravate this condition, since they are also effective against other parasites, such as *T. vivax*. In this case, the treated animals may present partial recovery from the infection and may not develop specific symptoms of the disease [8].

Bovine babesiosis is caused by the hemoprotozoa *Babesia bovis (B. bovis)* and *Babesia bigemina (B. bigemina)*, with the tick *Rhipicephalus microplus* representing the most important biological vector of these agents. The vector is distributed in tropical and subtropical regions, and Brazil is considered an enzootic country for tick-borne diseases. The economic losses of this disease are mainly due to increased mortality, reduced milk production, and low feed conversion [9,10].

To date, there have been no studies related to the prevalence or incidence of trypanosomiasis or babesiosis in dairy cows in Ceará [11]. Thus, the objective of this study was to evaluate the occurrence of and to collect epidemiological data relative to bovine trypanosomiasis and babesiosis, as well as to evaluate the possible impacts that the presence of these pathogens can cause in herds of dairy cows in the micro-region of the central hinterland of Ceará.

## 2. Materials and Methods

### 2.1. Ethics Committee

The research was carried out through a protocol submitted and approved by the Ethics Committee on the Use of Animals (CEUA) of the Federal Rural University of the Semi-Arid—UFERSA under protocol number 24/2021.

### 2.2. Study Area and Application of Questionnaires

**Figure 1:**
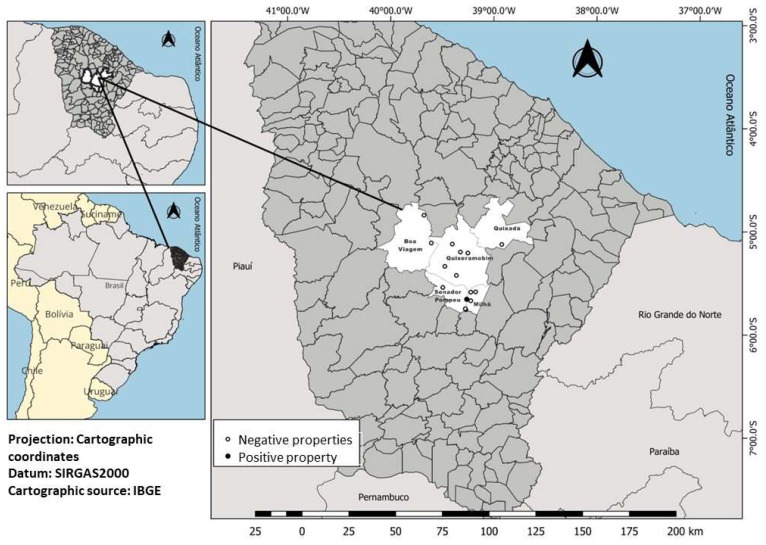
Georeferenced locations of the participating properties were utilized to assess the presence of *Trypanossoma vivax*, *Babesia bovis*, and *Babesia bigemina* in dairy cows in the hinterland of Ceará.

This study was conducted from November 2021 to March 2022 in the micro-region of hinterland of Ceará (Figure 1), more precisely in the municipality of Quixeramobim, where the largest milk production in the state is concentrated, located between latitude −5.19812 and longitude −39.2962, 5°11′53″ South, 39°17′46″ West, occupying a total area of 3276 km^2^. During most of the sample collections, the period was dry, with transition to a rainy season in the last month of sampling. The mean temperature in the region was 27 °C, with higher temperatures during the months of November and December (maximum 36 °C). Regarding precipitation, from November to February, the mean was 100–150 mm, and there was an increase in rainfall in March [12].

Information was collected by a questionnaire which aimed to obtain data from the owners and/or those responsible for the daily routine of the dairy herd regarding the rearing system, type of feed, type and management of mineral supplementation, and production aspects. In addition, the questionnaire allowed for the investigation of epidemiological factors such as the presence of horn flies or horseflies on the property, recent purchase of animals, use of oxytocin during milking, management of ectoparasites, and level of knowledge about trypanosomiasis and bovine babesiosis in the region.

A total of 246 blood samples were collected from dairy cows from 15 farms, all of which were evaluated for *T. vivax*; 160 were chosen for the evaluation of *B. bigemina* and *B. bovis*. The samples used for the detection of *B. bovis* and *B. bigemina* were chosen randomly, in a proportion of 8 to 11 samples from each property visited, varying according to the total number of animals in the herd. The scarcity of resources for research resulted in the need to use a smaller number of samples for the detection of babesia.

Approximately 9 mL of blood was collected from each animal, from the jugular or coccygeal vein, which was placed in sterile tubes containing ethylenediamine tetraacetic acid (EDTA). These were packed in thermal boxes and sent to the Laboratory of Diagnostics in Veterinary Clinical Pathology of the Federal Rural University of the Semi-Arid (PCVET-UFERSA).

### 2.3. Hematological Analysis

The mean corpuscular volume (MCV) was obtained by the microhematocrit method by centrifugation in capillaries at 11,500 rpm for 5 min and then read on a ruler.

### 2.4. Molecular Analysis

Molecular diagnosis for trypanosomiasis was performed at the Laboratory of Parasitic Diseases of the Department of Preventive Veterinary Medicine and Animal Health of the University of São Paulo (USP). Polymerase chain reaction (PCR) for *T. vivax* was performed using the protocol described by Cortez [13]. The added primers recognize a specific region of the parasite genetic material in which it is possible to observe the amplification of a fragment containing 210 bp of the TviSL (*T. vivax* Spliced leader) genomic region of the parasite. For the specific amplification of the TviSL gene, the primers TviSL1 (forward: 5′GCTCTCCAATCTTAACCCTA3′) and TviSL2 (reverse: 5′GTTCCAGGCGTGCAAACGTC3′) were added to the mixture. Amplifications were performed in a 50 mL mixture containing 200 mM dNTP, 20 mM primers TvSL1 and TvSL2, sample DNA (variable amount), and PCR buffer containing 1.5 mM MgCl2 and 2.5 U Taq DNA polymerase.

After the reagents were added, the samples were transferred to a thermal cycler, where the time was set at 35 cycles at 94 °C for 1 min for denaturation, 52 °C for 2 min for annealing and 72 °C for 3 min, followed by 72 °C for 10 min for final extension. After amplification, the products were added to glass plates for electrophoresis containing a molecular weight standard and positive and negative control samples (distilled water), in 1.5% agarose gel and ethidium bromide. For all reactions, known positive DNA samples for the agent under investigation were used as positive controls [13], while DEPC water was used as a negative control. Reading was performed on a ultralight (UV) transilluminator, and sample amplifications were compared with the molecular weight standard, positive and negative control version 5.0 (Prism Software^®^, Irvine, CA, USA).

The molecular detection of *Babesia* sp. was performed at the Laboratory of Morphophysiopharmacology of the Department of Health Sciences of the UFERSA, Mossoró campus. For the detection of *B. bigemina* and *B. bovis* DNA, 8 to 11 animals from each farm were randomly selected for a total of 160 samples. Samples were subjected to genomic DNA extraction using the commercial PureLink^®^ Genomic DNA Mini Kit (Thermo Fisher Scientific, Waltham, MA, USA) according to the manufacturer’s recommendations. After extraction, the genomic material was quantified in a spectrophotometer (NanoDrop™ Lite, Thermo Scientific) and the samples stored at −20 °C until PCR.

For the detection of *B. bigemina* and *B. bovis* DNA, primer oligonucleotides validated in previously published papers were used. PCR was directed to the *B. bigemina* SS rRNA gene using primers GAU6-R (CCACGCTTGAAGCACAGGA) and GAU7-F (GTTGGGTCTTTTCGCTGGC) and to the *B. bovis* SS rRNA gene using primers GAU9-F (CTGTCGTACCGTTGGTTGAC) and GAU10-R (CGCACGGACGGAGACCGA). PCRs were performed in a final volume of 25 μL, containing 12.5 μL of Hot Start Taq Pol Master Mix (2X) (Cellco^®^, São Carlos, Brazil), 1 μL of each primer (10 mM), 9.5 μL of DEPC water and 1 μL of sample. For all reactions, known positive DNA samples for the agents under investigation were used as positive controls [9], while DEPC water was used as a negative control.

The PCR amplified products were submitted to electrophoresis in 1.5% agarose gel, in 1X Tris-Borate-EDTA (TBE) buffer, using ethidium bromide as DNA staining agent for 45 min at 120 volts. To determine the size of the amplified products, a 100 bp molecular weight marker (Ludwig^®^ biotechnology, São Paulo, Brazil) was used following the manufacturer’s recommendations. After electrophoresis, the gel was visualized in a UV light transilluminator (Proteinsimple^®,^ San Jose, CA, USA) to determine the size of the amplified product. The samples were considered positive for the detection of *B. bigemina* SS rRNA gene and *B. bovis* SS rRNA gene when amplified products with an approximate size of 685 bp and 541 bp, respectively, were detected [14].

### 2.5. Statistical Analysis

The variables infection by *B. bovis* and *B. bigemina*, coinfection by both parasites, purchase of recent animals, tick control, and mean corpuscular volume of red blood cells were selected and analyzed for normality by the Kolmogorov–Smirnov test. The Mann–Whitney U-test was used for the comparison of independent and continuous samples (mean corpuscular volume vs. *B. bovis* or *B. bigemina* infection or co-infection) and the chi-squared test was chosen for the inferential analysis of categorical data (purchase of recent animals or tick control vs. *B. bovis* or *B. bigemina* infection or co-infection). Statistical significance was set at *p* ≤ 0.05.

## 3. Results

**Figure 2:**
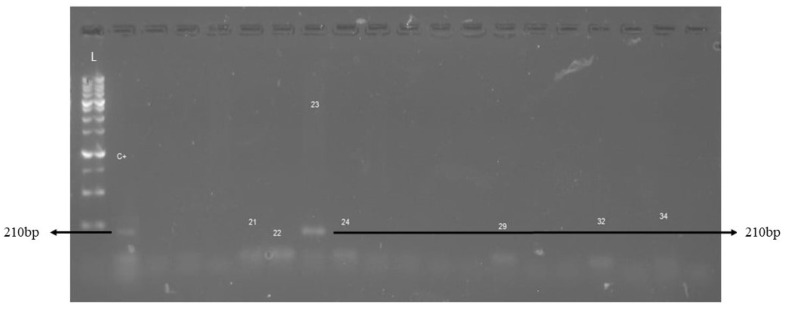
Detection of the *T. vivax* cdCatL-like gene by conventional PCR in dairy cows from the central hinterland of Ceará, 2021/2022. L: molecular weight marker, 100 bp. C+: positive control; Sample 23: amplification of a fragment of approximately 210 bp specific to the cdCatL-like gene of *T. vivax*.

**Figure 3:**
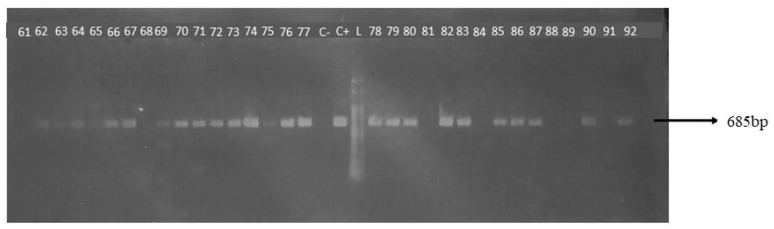
Detection of *B. bigemina* SS rRNA gene by conventional PCR in dairy cows from the central hinterland of Ceará, 2021/2022. L: molecular weight marker, 100 bp. C+: positive control; C−: negative control; Samples 62–64, 66, 67, 69–80, 82, 83, 85–87, 90 and 92: amplification of a fragment of approximately 685 bp specific to the *B. bigemina* SS rRNA gene.

**Figure 4:**
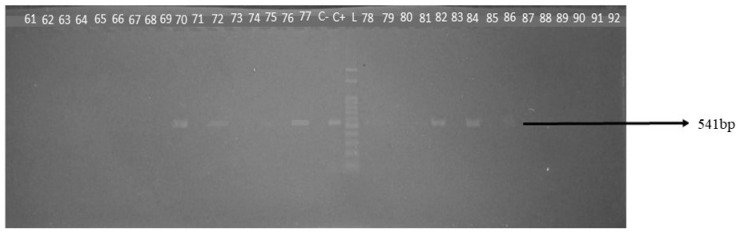
Detection of the *B. bovis* SS rRNA gene by conventional PCR in dairy cows from the central hinterland of Ceará, 2021/2022. L: molecular weight marker, 100 bp. C+: positive control; C−: negative control; Samples 70, 72, 75, 77, 78, 82, 84, 86: amplification of a fragment of approximately 541 bp specific to the *B. bovis* SS rRNA gene.

The occurrence of the pathogens investigated in this study was 0.4% (1/246) for *T. vivax* (Figure 2), 20.62% (33/160) for *B. bigemina* (Figure 3), and 11.87% (19/160) for *B. bovis* (Figure 4).

Based on the information collected in the questionnaires, it was possible to characterize the properties that participated in this study. All properties had dairy production as their main subsistence activity. The average milk production of the properties was 1005 L of milk per day, with the property with the highest production having an average of 5200 L per day and the lowest production being 150 L per day.

After evaluating the questionnaire, it was found that, in relation to the presence of vectors, 73.33% (11/15) of the producers answered that there was a presence of horn flies and/or horseflies on the property, while 53.33% (8/15) reported having recently purchased new animals, 80.00% (12/15) confirmed having recurrent reproductive problems in the herd, such as cases of abortion and repeated estrus, and 66.66% (10/15) stated that they used oxytocin during the milking of the animals. Regarding the management of ectoparasites, all 15 producers or employees stated that they carry out control practices using ectoparasiticides in the form of pour-on.

When asked about their knowledge of diseases caused by hemoprotozoa, eleven producers reported knowing about trypanosomiasis, three said they had already had cases of the disease among their animals, and one of them had been undergoing “preventive” treatment for more than 2 years. In relation to babesiosis, all 15 of the producers knew of the disease.

**Figure 5:**
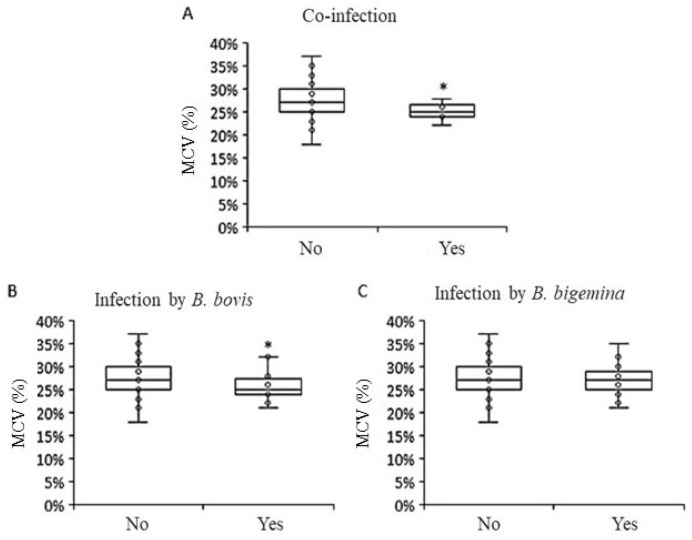
Correlation between independent samples and percentage of mean corpuscular volume for co-infection by *B. bovis* and *B. bigemina* (**A**) (*p* = 0.04) and infection only by *B. bovis* (**B**) (*p* = 0.05) and *B. bigemina* (**C**) (*p* = 0.50) of dairy cows from the central hinterland of Ceará. * = difference *p* ≤ 0.05 between groups evaluated. MCV = mean corpuscular volume; No = non-infected animals; Yes = animals infected by parasites.

From the questionnaires, it was also noted that the farm with a positive case of *T. vivax* had previously experienced the disease; reported a low incidence of horn flies or horseflies; purchased animals from external sources within the past 6 months; used oxytocin during milking; and had cases of abortion, repeated estrus, and neurological symptoms within the herd. Regarding the evaluation of mean corpuscular volume, it was possible to observe an average of 27.74% among all the animals evaluated, with a variation of 18% to 37%. Among these samples, it was found that 9.75% (24/246) of the cows had a value below 24%. When comparing the mean corpuscular volume values with cases of co-infection of *B. bovis* and *B. bigemina*, it was found that there was a reduction in the percentage of mean corpuscular volume of red blood cells (*p* < 0.05) (Figure 5A). However, when analyzing the infections separately, it was shown that the reduction in mean corpuscular volume occurred in animals infected with *B. bovis* (*p* = 0.05) and not in those with only *B. bigemina* infection (*p* > 0.05) (Figure 5B and 5C, respectively).

In order to investigate whether there was an association between tick control and the occurrence of infection, the following variables were analyzed: tick control vs. infection by *B. bovis* and *B. bigemina* or co-infection by both parasites (Figure 5). However, no significant differences were found in the evaluations, indicating that tick control, or the lack thereof, did not influence the presence or absence of babesiosis. Additionally, the influence of recent animal purchases on the occurrence of *B. bovis*, *B. bigemina*, or co-infection on the property was assessed. No significant differences were observed here either, demonstrating that recent animal purchases had no effect on the incidence of babesiosis.

## 4. Discussion

In the present study, only one animal was found to have DNA for *T. vivax*; however, from the information collected in the questionnaires, it is possible to identify epidemiologically important factors that may facilitate the transmission of the parasite to healthy animals, such as recent and recurrent purchases of animals and the use of the same needle and syringe for the application of oxytocin.

The practice of the intravenous application of oxytocin to lactating cows during milking is common and performed in order to quickly increase milk production, reducing the time needed to milk the same number of animals [15]. Although it seems to bring beneficial results for milk production, it predisposes the animals to the transmission of several diseases and can be considered the main form of infection by *T. vivax* [16].

Despite the existence of a study reporting the presence of *T. vivax* in Ceará [17], this was carried out in only one herd and with the initial objective of evaluating the clinical changes and reproductive rates of infected dairy cows compared to healthy ones. Thus, the present study is the first to evaluate the occurrence of *T. vivax* in properties located in the microregion of the central hinterland of Ceará.

Although most of the producers in the present study reported problems related to reproduction on their properties, it can be verified that these cases were not correlated with the presence of trypanosomiasis. However, several other disorders that usually affect herds, such as babesiosis, can cause similar symptoms. Because of this, it is believed that bovine trypanosomiasis is underdiagnosed in Brazil and may be further aggravated by the use of drugs to treat babesiosis and anaplasmosis before a definitive diagnosis, as these drugs have some pharmacological efficacy on trypanosomes, which can lead to a partial recovery of the animal by reducing parasitemia, causing some misunderstanding about the real cause of the problem [8].

Hemotropic pathogens can contribute to morbidity, mortality, and infertility in cattle and can occur concomitantly with *T. vivax* infections, which has a great impact on productivity. Babesiosis was diagnosed in most of the properties visited, and some animals had co-infection of *B. bovis* and *B. bigemina*. Usually, *B. bigemina* infection is characterized by a low level of parasitemia, while *B. bovis* causes high parasitemia, in addition to more severe clinical signs, such as fever, depression, hemolytic anemia, hemoglobinuria, and icterus [18].

Despite the occurrence of babesiosis, none of the positive animals had any clinical symptoms or reduction in mean corpuscular volume that could be related to the presence of the disease. This may be a consequence of the greater resistance of the animals, because of the good body score that they presented, adequate nutritional management, and the generalized tick control and drug treatment of the affected cattle, which has become a common practice adopted by producers. However, 9.75% of the evaluated cows, which were negative for babesiosis, had a mean corpuscular volume value below 24%, indicating the probable presence of other pathogens or problems in these animals.

The fact that there are animals carrying *Babesia* sp. without clinical manifestation can cause even greater problems for the producer and for the health of the animals; because of the lack of symptoms, there is no incentive for treatment. In addition, in cases of *B. bovis* infection, it is believed that animals remain carriers for life. Meanwhile, animals that have already recovered from *B. bigemina* infection may eliminate the infection and have a reduction in antibody titer, which may remain below the negative limit and favor a new infection, but in a subclinical form, or they may end up becoming carriers of the parasite, which facilitates the further spread of the disease as they become a source of infection for carrier ticks [19,20].

The indiscriminate use of antiparasitic drugs to combat ticks should also be discussed with producers in order to avoid the development of resistance by ticks, making it increasingly complicated to treat infected animals that really need more efficient drugs [21]. Usually, the most common method of control is from the use of ivermectin in the herd, but it has already been reported that tick populations from the Brazilian semi-arid region present resistance to the drug, in addition to being able to develop cross-resistance to moxidectin [22].

Regarding the medication used to treat cases of trypanosomiasis, many of the producers demonstrated knowledge of this, since veterinarians from the region had visited the properties in previous years for the diagnosis and indication of treatment for sick animals. This was due to the increasing number of cases detected in 2019 [11], but there are no reports on the total number of properties, animals, and samples analyzed or the number of positive cases detected.

In this study, on one of the properties, the producer declared the use of the drug imidocarb dipropionate as a preventive measure, even though he did not have positive animals in his herd. However, manufacturers do not indicate the drug for this purpose, nor for outbreak cases. The inappropriate use of the drug can lead to the development of protozoan strains resistant to this and other compounds, such as isometamidium and diminazene diaceturate [2]. In addition, it is possible that the parasite can escape trypanocidal drugs by invading other areas of the animal’s body where the drug cannot achieve adequate plasma levels to eliminate it, such as adipose tissue [23], the skin [24], and the central nervous system [25].

The semi-arid region can be considered an area of enzootic instability for both trypanosomiasis and babesiosis and bovine anaplasmosis. This is a consequence of long periods of dryness with high temperatures that make the region less favorable for the development of vectors during most of the year, making it impossible for animals to develop active immunity. Thus, outbreaks can occur when the parasite comes from other regions and multiplies during the rainy season [7,26].

The damage caused by the presence of both diseases in animals, reflected in decreased milk production and reproductive problems, as well as the high cost of the drugs that are needed, can make the upkeep of dairy herds unviable if there is no effective control of important factors in the transmission and care related to the arrival of external pathogens. In order to provide a more complete economic evaluation, the epidemiological situation in the region where the cattle are located, curative or prophylactic treatments, and veterinary assistance must be taken into account [27].

Regarding bovine babesiosis, although all 15 properties had positive cases in the herd, none of the animals evaluated showed clinical manifestations of the disease, possibly due to the good nutritional management maintained on the properties, the relatively efficient control to avoid the spread of ticks, and the climatic conditions of the region. It is desirable for cattle to be exposed to *Babesia*-infected vectors when they are still calves, between 7 and 9 months of age, since they present resistance to the clinical effects of the disease at this stage, in addition to developing immunity against the parasite [28].

## 5. Conclusions

The agents *T. vivax*, *B. bigemina*, and *B. bovis* were identified in animals from the participating farms. The detection of an animal positive for *T. vivax* can be interpreted as a warning to producers, who should aim to avoid practices that facilitate the spread of these agents among animals. In the context of babesiosis, none of the animals molecularly identified as positive exhibited clinical symptoms, suggesting a potential immunity of the herds to these parasites. The presence of the identified agents may be linked to reproductive issues, reduced productivity, and animal deaths.

## Data Availability

The original contributions presented in the study are included in the article, further inquiries can be directed to the corresponding author.
